# Effects of urea plus nitrate pretreated rice straw and corn oil supplementation on fiber digestibility, nitrogen balance, rumen fermentation, microbiota and methane emissions in goats

**DOI:** 10.1186/s40104-019-0312-2

**Published:** 2019-01-23

**Authors:** Xiumin Zhang, Rodolfo F. Medrano, Min Wang, Karen A. Beauchemin, Zhiyuan Ma, Rong Wang, Jiangnan Wen, Lukuyu A. Bernard, Zhiliang Tan

**Affiliations:** 1 Key Laboratory for Agro-Ecological Processes in Subtropical Region, National Engineering Laboratory for Pollution Control and Waste Utilization in Livestock and Poultry Production, Institute of Subtropical Agriculture, Chinese Academy of Sciences, Changsha, 410125 Hunan China; 2 University of the Chinese Academy of Sciences, Beijing, 100049 China; 3 College of Veterinary Science and Medicine, Central Luzon State University, Science City of Muñoz, 3120 Nueva Ecija, Philippines; 4 College of Animal Science and Technology, Hunan Agricultural University, Changsha, 410128 People’s Republic of China; 5 Agriculture and Agri-Food Canada, Lethbridge Research and Development Centre, Lethbridge, Alberta T1J 4B1 Canada; 6 International livestock Research Institute, POB 30709, Nairobi, 00100 Kenya

**Keywords:** Dissolved hydrogen, Methane, Nitrate, Oil, Rumen fermentation, Urea

## Abstract

**Background:**

Urea pretreatment is an efficient strategy to improve fiber digestibility of low quality roughages for ruminants. Nitrate and oil are usually used to inhibit enteric methane (CH_4_) emissions from ruminants. The objective of this study was to examine the combined effects of urea plus nitrate pretreated rice straw and corn oil supplementation to the diet on nutrient digestibility, nitrogen (N) balance, CH_4_ emissions, ruminal fermentation characteristics and microbiota in goats. Nine female goats were used in a triple 3 × 3 Latin Square design (27 d periods). The treatments were: control (untreated rice straw, no added corn oil), rice straw pretreated with urea and nitrate (34 and 4.7 g/kg of rice straw on a dry matter [DM] basis, respectively, UN), and UN diet supplemented with corn oil (15 g/kg soybean and 15 g/kg corn were replaced by 30 g/kg corn oil, DM basis, UNCO).

**Results:**

Compared with control, UN increased neutral detergent fiber (NDF) digestibility (*P* < 0.001) and copies of protozoa (*P* < 0.001) and *R. albus* (*P* < 0.05) in the rumen, but decreased N retention (−21.2%, *P* < 0.001), dissolved hydrogen concentration (−22.8%, *P* < 0.001), molar proportion of butyrate (−18.2%, *P* < 0.05), (acetate + butyrate) to propionate ratio (*P* < 0.05) and enteric CH_4_ emissions (−10.2%, *P* < 0.05). In comparison with UN, UNCO increased N retention (+34.9%, *P* < 0.001) and decreased copies of protozoa (*P* < 0.001) and methanogens (*P* < 0.001). Compared with control, UNCO increased NDF digestibility (+8.3%, *P* < 0.001), reduced ruminal dissolved CH_4_ concentration (−24.4%, *P* < 0.001) and enteric CH_4_ emissions (−12.6%, *P* < 0.05).

**Conclusions:**

A combination of rice straw pretreated with urea plus nitrate and corn oil supplementation of the diet improved fiber digestibility and lowered enteric CH_4_ emissions without negative effects on N retention. These strategies improved the utilization of rice straw by goats.

## Background

Rice straw is an abundant crop residue in rice producing areas, such as China and Southeast Asia. However, rice straw has low nutritive value, which limits its wide and efficient use in ruminant diets, because of the high content of indigestible structural polysaccharides and low crude protein (CP) content [1]. Enteric methane (CH_4_) resulting from ruminants is an important source of greenhouse gas and represents a loss of 2–14% of dietary energy [2]. Feeding straw results in greater CH_4_ emissions per unit of feed digested than high quality forage, because it has a slow passage rate and prolonged residency time in the rumen [3]. Before promoting the use of rice straw in ruminant diets, strategies are needed to improve its digestibility and decrease its CH_4_ emissions.

Urea can be used to pretreat rice straw to enhance its nutritional quality by destroying fiber structure and increasing non-protein nitrogen (N) content [1], leading to increased ruminal fiber digestibility [4, 5]. Nitrate supplementation has been identified as a possible strategy to reduce CH_4_ emissions [6, 7], because nitrate reduction to ammonia incorporates metabolic hydrogen ([H]) away from methanogenesis [8]. Urea plus nitrate pretreatment has been reported to increase degradation and reduce CH_4_ production of rice straw in vitro [9], and further verification of these combined effects in vivo is needed.

Oil supplementation is another option to decrease enteric CH_4_ emissions from ruminants and improve final quality of product (i.e., milk and meat). Oil supplementation increases energy density of the diet, provides additional unsaturated fatty acids and decreases numbers of protozoa and associated methanogens, but may also decrease ruminal organic matter (OM) fermentation [3]. Additionally, inhibition of ruminal protozoa can decrease microbial N cycling in the rumen [10] and increase the flow of microbial protein from the rumen to the small intestine [11], leading to an improvement of N utilization. Thus, oil supplementation of diets containing urea plus nitrate pretreated rice straw may further help to improve efficiency of dietary N use in ruminants.

In the present study, we hypothesized that pretreatment of rice straw with urea plus nitrate would enhance fiber digestibility and inhibit CH_4_ production, and supplementation of corn oil to a diet containing the pretreated straw would further exert additional beneficial effects on dietary N use efficiency and CH_4_ mitigation. To test these hypotheses, we used goats as the experimental animal, and measured diet digestibility, N balance, CH_4_ emissions, rumen fermentation, and selected microorganisms.

## Methods

### Urea plus nitrate pretreated rice straw

The rice straw originated from Jiangxi province of China. Whole plant material was obtained and thrashed to remove the grain and the straw was sun-cured and chopped into pieces of approximately 3 cm in length. Straw was pretreated with urea (34 g/kg straw dry matter [DM]) and 6 g/kg DM of ammonium nitrate (to supply 4.7 nitrate g/kg straw DM) for the urea plus nitrate treatment (UN). The pretreatment procedure was fully described by Zhang et al. [9]. Briefly, urea and ammonium nitrate were dissolved in water (40%, *w/v*) and sprayed onto the straw to achieve the appropriate concentrations. The pretreated straw was then placed into sealed 200 L plastic vessels and incubated at 15 ± 3.0 °C (means ± SD) for 4 weeks. After incubation, the straw was sun-cured for feeding.

### Experimental design, goats, and diets

Nine female Liuyang Black goats (a local breed in southern China, 1 year of age) with body weight of 19.0 ± 1.22 kg (mean ± SD) at the start of the experiment were used. The experiment was a triple 3 × 3 Latin Square design with 3 dietary treatments (Table 1). The control diet was formulated to meet appropriately 1.2 to 1.3 times the digestible energy (DE) and CP requirements of non-pregnant female goats according to Zhang and Zhang [12], and consisted of 50% rice straw and 50% concentrate (Table 1). The UN diet was formulated by replacing untreated rice straw with UN pretreated rice straw. The combination of UN and corn oil supplementation (UNCO) diet was formulated by replacing soybean meal (15 g/kg of dietary DM) and corn grain (15 g/kg of dietary DM) with corn oil (30 g/kg of dietary DM). Goats were fed individually at 07:30 and 17:30 h with equal portions of feed offered at each meal.

**Table 1:** Ingredients and chemical composition of the diets (DM basis)

Items	Treatment^1^		
	Control	UN	UNCO
Ingredients, g/kg			
Untreated rice straw^2^	500	–	–
Urea plus nitrate pretreated rice straw^3^	–	500	500
Soybean meal^4^	80.0	80.0	65.0
Corn grain	292	292	277
Wheat bran	98.0	98.0	98.0
CaCO_3_	1.00	1.00	1.00
CaH_2_PO_4_	4.00	4.00	4.00
NaCl	5.00	5.00	5.00
Premix	20.0	20.0	20.0
Corn oil^5^	–	–	30.0
Chemical composition, g/kg			
OM	910	910	921
CP	105	121	119
NDF	468	458	440
ADF	271	266	257
GE, MJ/kg	16.2	16.3	16.3
EE	10.2	10.2	40.2
Nitrate	–	2.33	2.33

^1^*UN* urea plus nitrate pretreated rice straw, *UNCO* combination of UN and corn oil

^2^Rice straw contained 898 g OM, 34.4 g CP, 766 g NDF and 470 g ADF per kg DM

^3^Urea plus nitrate pretreated rice straw contained 898 g OM, 68.1 g CP, 744 g NDF and 458 g ADF per kg DM

^4^Contained 442 g CP per kg DM

^5^The corn oil was supplied by Wilmar International Ltd., Wuhan, China. Composed of 15% saturated fatty acids, 32% monounsaturated fatty acids and 53% polyunsaturated fatty acids

The experiment consisted of 3 periods, each with 27 d including 14 d for diet adaptation, 5 d for collecting feces and urine, 6 d for measuring CH_4_ emissions and 2 d for rumen sampling. All goats were housed in individual metabolism cages and had free access to drinking water.

### Apparent total-tract digestibility and N balance

Total feces and urine were collected and weighed twice daily from d 15 to 19. A subsample (~ 1%) of feces and urine from each animal was obtained at each collection time and frozen immediately at − 20 °C, and another subsample (~ 1%) was acidified using 10% (*w/w*) H_2_SO_4_ to prevent N loss and then frozen immediately at − 20 °C. The subsamples were then individually combined by day and goat within period. The acidified samples were used for total N analysis, whereas non-acidified samples were used for other chemical analysis.

The N balance, including N excretion and retention, was calculated using the following equations:

N excretion (g/d) = fecal N (g/d) + urinary N (g/d).

N excretion (%) = [N excretion (g/d) / N intake (g/d)] × 100%.

N retention (g/d) = N intake (g/d) – N excretion (g/d).

N retention (%) = [N retention (g/d) / N intake (g/d)] × 100%.

### Enteric methane emission

After total collection, CH_4_ emissions were measured for 2 d per goat by moving each goat into a respiration chamber as described by Wang et al. [13]. As only three chambers were available, it took 6 d (from d 20 to 25) to complete the measurements for all goats. Nine goats were assigned to 3 blocks, with each block containing 3 goats (1 goat from each treatment). The goats in each block were assigned to the same chamber and 1 goat from each block was used for each of the 2-day sequential measurements. The goats were restrained but had free access to a feed bin and drinking water within the chamber. On average, the flow rate of air was maintained at 40 m^3^/h. The CH_4_ concentration in outlet gas from each chamber and ambient gas were measured using an Ultraportable Greenhouse Gas Analyzer (MIU-374-8, Los Gatos Research, San Jose, CA 95134, USA). The cycling time to separately measure CH_4_ concentration from the 3 chambers was 30 min, with 8 min for each chamber gas and 6 min for ambient gas. Calibrations were done by discharging purified CH_4_ into each chamber at 25 cm^3^/min controlled by a gas flow meter (C100L-CRWE-DD, SIYA Detection Instrument Co., Ltd., Shanghai, China). The gas flow data from both the gas flow meter and chamber were calculated and compared, and then the data for the chamber were adjusted to 100% recovery according to the data of the gas flow meter. The chambers were opened twice a day at 07:30 and 17:30 h to deliver the diets, and the chambers were cleaned before the morning feeding.

### Rumen sampling

Rumen contents were collected at 0, 2.5 and 6 h after morning feeding on 2 consecutive days (d 26 and d 27) using an oral stomach tube according to Wang et al. [13]. About 150 mL of rumen contents were rapidly collected after discarding the initial 100 mL of rumen contents. Two subsamples (15 mL each) were immediately frozen at − 80 °C in liquid nitrogen for DNA extraction and subsequent microbial analysis. Two other subsamples (35 mL each) were immediately transferred into 50-mL plastic syringes for measuring dissolved hydrogen (dH_2_) and dissolved CH_4_ (dCH_4_) concentrations according to the procedures outlined by Wang et al. [14]. About 10 mL of rumen contents were used for the measurement of ruminal pH using a portable pH meter (Starter 300; Ohaus Instruments Co. Ltd., Shanghai, China). An aliquot of 2.5 mL of rumen contents was centrifuged at 15,000×*g* for 10 min at 4 °C, and supernatant (1.5 mL) was transferred into tubes containing 0.15 mL of 25% (*w/v*) metaphosphoric acid, vigorously hand-shaken and stored at − 20 °C for subsequent determination of volatile fatty acid (VFA) and ammonia concentration.

### Sample analyses

The samples of feed, orts and feces were dried in a forced-air oven at 65 °C for 48 h, ground through a 0.25-mm screen (WJX-100, Shanghai, Yuanwo Company) and analyzed in triplicate for DM, OM, ether extract (EE), neutral detergent fiber (NDF), acid detergent fiber (ADF), N content and gross energy (GE). The DM (method 930.15), OM (method 942.05), EE (method 963.15) and N (method 970.22) were analyzed according to published methodologies [15]. Neutral detergent fiber and ADF were assayed according to the method of Van Soest et al. [16], and expressed inclusive of residual ash. Heat stable α-amylase was added during the NDF analysis. Gross energy was determined using an isothermal automatic calorimeter (5E-AC8018, Changsha Kaiyuan Instruments Co., Ltd., China). Individual VFA concentrations were measured using a gas chromatograph (Agilent 7890, Palo Alto, CA, USA) according to the procedure described by Wang et al. [14]. Ammonia-N concentration was measured by UV–Vis spectrophotometer (Shimadzu UV-2450, Kyoto, Japan) according to the protocol described by Chaney and Marbach [17].

### Microbial analyses

Rumen samples taken 2.5 h after the morning feeding were freeze-dried (CHRIST RVC2–25 CDPIUS, Marin Christ CO., Ltd., Osterode, Germany) for microbial analysis. The DNA was extracted with repeated bead beating plus column purification as described by Yu and Morrison [18], and eluted by 300 μL TE buffer (Tris 10 mmol/L, EDTA 1 mmol/L, pH = 8.0). The quality and quantity of DNA were measured based on absorbance at 260 and 280 nm using a NanoDrop ND-2000 (NanoDrop Technologies Inc., Wilmington, USA).

The 16S rRNA gene V3-V4 hypervariable regions of bacteria genomic DNA were used for PCR amplification with the primers 5′- ACTCCTACGGGAGGCAGCAG-3´ (338F) and 5′- GGACTACHVGGGTWTCTAAT-3′ (806R). The PCR reactions were performed in triplicate using a 20-μL mixture containing 0.8 μL of each primer, 10 ng of template DNA, 2 μL 2.5 mmol/L dNTPs, 0.4 μL of FastPfu polymerase (Transgen, Beijing, China) and 4 μL 5 × FastPfu Buffer (Transgen, Beijing, China). The thermal cycling programing was performed as follows: initial denaturation step, 95 °C, 3 min; denaturation, 27 cycles, 95 °C, 30 s; annealing, 55 °C, 30 s; elongation, 72 °C, 45 s; and final extension, 72 °C, 10 min. The PCR products were excised from 2% agarose gels and purified using a QIAquick Gel extraction kit (Qiagen, Hilden, Germany). Amplicons from each reaction mixture were quantified fluorometrically, normalized and pooled at equimolar ratios based on the concentration of each amplicon. Amplicons were sequenced with the Illumina MiSeq platform (PE300, Majorbio Bio-Pham Technology, Shanghai, China). Quality control of the sequence reads was performed using MOTHUR v.1.39.5 [19] following the protocol described by Kozich et al. [20]. The high-quality reads were clustered into operational taxonomic units (OTU) at 97% similarity using Usearch v.7.0 [21]. Representative sequences defined by abundance from each OTU were identified using PyNAST [22] against the SILVA database for bacteria [23]. Taxonomy analysis was performed using the Ribosomal Database Project classifier v.11.1 [24] with a minimum support threshold of 80%. Alpha diversity analyses were generated, including observed species (Sobs), Chao, Ace, Shannon-Weiner, and Simpson’s indices. All 16S rRNA gene sequences were deposited into the NCBI Sequence Read Archive under accession number SRP155601.

Real-time quantitative PCR (qPCR) was performed according to the procedures described by Jiao et al. [25]. Briefly, the plasmid DNA containing exact 16S or18S rRNA gene inserts were used to make a standard curve for selected microbial groups. All standard curves met the following requirements (*R*^2^ > 0.99, 90% < *E* < 120%). The protozoa, fungi, total bacteria, total methanogens, and four selected bacteria were studied. *Fibrobacter succinogenes* (*F. succinogenes*), *Ruminococcus albus* (*R. albus*) and *Ruminococcus flavefaciens* (*R. flavefaciens*) were selected because of their predominant role in fiber digestion [26]; and *Selenomonas ruminantium* (*S. ruminantium*) was selected because it plays a role in nitrate and nitrite reduction [27]. Forward and reverse primers of the selected microbial groups are shown in Table 2.

**Table 2:** Primers for qPCR assay

Microbial Species	Primer Sets (5′→3′)	Product size, bp	References
Protozoa	F: GCTTTCGWTGGTAGTGTATT; R: CTTGCCCTCYAATCGTWCT	223	[47]
Fungi	F:GAGGAAGTAAAAGTCGTAACAAGGTTTC; R:CAAATTCACAAAGGGTAGGATGATT	121	[48]
Bacteria	F: CGGCAACGAGCGCAACCC; R: CCATTGTAGCACGTGTGTAGCC	146	[48]
Methanogens	F: GGATTAGATACCCSGGTAGT; R: GTTGARTCCAATTAAACCGCA	192	[49]
Selected groups of bacteria			
*Fibrobacter succinogenes*	F:GTTCGGAATTACTGGGCGTAAA; R: CGCCTGCCCCTGAACTATC	121	[48]
*Ruminococcus albus*	F:CCCTAAAAGCAGTCTTAGTTCG; R: CCTCCTTGCGGTTAGAACA	176	[50]
*Ruminococcus flavefaciens*	F: GAACGGAGATAATTTGAGTTTACTTAGG; R: CGGTCTCTGTATGTTATGAGGTATTACC	132	[48]
*Selenomonas ruminantium*	F: CAATAAGCATTCCGCCTGGG; R: TTCACTCAATGTCAAGCCCTGG	138	[51]

### Statistical analyses

The statistical analysis was performed using the mixed linear model procedure of SPSS 19.0 (Chicago, IL, USA). Fermentation and CH_4_ emissions data were averaged to obtain a mean value for the 2 consecutive days before further analysis. For the data of CH_4_ emissions, digestibility, qPCR, relative abundance and alpha diversity indexes estimated from 16S rRNA gene library sequences, the model used included treatment (*n* = 3) as a fixed effect, and period (*n* = 3) and animal (*n* = 9) as random effects. When sampling time was included, the model included dietary treatment (*n* = 3) and the interaction of treatment and sampling time as a fixed effect, sampling time (*n* = 3) as a repeated measurement, and animal (*n* = 9) and period (*n* = 3) as random effects. When significant differences were found, a multiple comparison was conducted to elucidate differences between two particular treatments, and *P*-values were adjusted using the Bonferroni method. Statistical significance was declared at *P* ≤ 0.05, with a tendency towards significance declared at 0.05 < *P* ≤ 0.10.

## Results

Goats fed UN diet had a greater dry matter intake (DMI) than control goats (*P* = 0.03, Table 3), but DMI of goats fed UNCO was similar to those fed UN and control diets. Total-tract digestibility of OM (*P* < 0.001) and NDF (*P* < 0.001) was greater for goats fed UN or UNCO compared with those fed control diet. However, goats fed UNCO had greater total-tract digestibility of GE than those fed UN (*P* < 0.001) or control diets (*P* < 0.001).

**Table 3:** Effect of urea plus nitrate pretreated rice straw (UN) and combination of UN and corn oil supplementation (UNCO) on apparent total-tract digestibility, N balance and methane emissions in goats

Items	Treatment			SEM	*P* - value
	Control	UN	UNCO		
DMI, g/d	484^b^	501^a^	496^ab^	4.2	0.03
Apparent total-tract digestibility, g/kg					
DM	656^b^	666^ab^	674^a^	7.0	0.05
OM	688^b^	711^a^	720^a^	10.5	< 0.001
NDF	542^b^	577^a^	587^a^	11.1	< 0.001
ADF	530	539	538	10.9	0.62
CP	631	634	638	14.0	0.76
GE	629^b^	641^b^	668^a^	12.1	< 0.001
Nitrogen balance					
N intake, g/d	8.41^c^	9.81^a^	9.13^b^	0.051	< 0.001
Urinary N, g/d	2.94^b^	4.35^a^	3.32^b^	0.198	< 0.001
Urinary N to N intake ratio, %	35.0^b^	44.3^a^	34.4^b^	2.19	< 0.001
Fecal N, g/d	3.10^b^	3.60^a^	3.30^b^	0.141	< 0.001
Fecal N to N intake ratio, %	36.9	36.6	36.2	1.39	0.77
N excretion, g/d	6.05^b^	7.95^a^	6.62^b^	0.238	< 0.001
N excretion to N intake ratio, %	71.9^b^	81.0^a^	72.5^b^	2.19	< 0.001
N retention, g/d	2.36^a^	1.86^b^	2.51^a^	0.208	< 0.001
N retention to N intake ratio, %	28.1^a^	19.1^b^	27.5^a^	2.40	< 0.001
Methane emissions					
g/d	9.91^a^	9.24^ab^	8.92^b^	0.779	0.04
g/kg DMI	20.6^a^	18.5^b^	18.0^b^	1.59	0.03
g/kg OM digested	32.8^a^	28.5^b^	27.3^b^	2.32	< 0.001
g/kg NDF digested	76.3^a^	61.2^b^	63.7^b^	4.44	< 0.001
% of GE intake	6.41^a^	5.69^b^	5.32^b^	0.481	0.004

^a-c^Means with different superscripts within a row are significantly different (*P* ≤ 0.05)

Goats fed UN had greater N intake (+16.6%, *P* < 0.001) and total N excretion (+31.4%, *P* < 0.001), and less N retention (−21.2%, *P* < 0.001) than those fed control (Table 3). In comparison with goats fed UN, those fed UNCO consumed less N (−6.9%, *P* < 0.001) and excreted less N (−16.7%, *P* < 0.001), and retained a greater amount of N (+34.9%, *P* < 0.001). Compared with control, goats fed UNCO had greater N intake (+8.5%, *P* < 0.001), but did not differ in N excretion and retention.

Compared with control, goats fed UN diet had less (*P* < 0.05) daily CH_4_ emission expressed as g/kg DMI, g/kg OM digested, g/kg NDF digested and % of GE intake (Table 3). Goats fed UNCO also had less (*P* < 0.05) CH_4_ emissions expressed as g/d than those fed the control diet.

Goats fed UN had greater mean ruminal pH (*P* < 0.001) and ammonia-N concentration (+30.8%, *P* < 0.05), lower dH_2_ concentration (−26.8%, *P* < 0.001), molar proportions of butyrate (−18.2%, *P* < 0.001), isobutyrate (*P* < 0.001) and valerate (*P* < 0.05), and (acetate + butyrate) to propionate ratio (*P* < 0.05) in comparison with those fed control (Table 4). Corn oil supplementation to the UN diet decreased mean pH (*P* < 0.001), and increased molar proportions of butyrate (+31.7%, *P* < 0.001) and valerate (*P* < 0.05). Goats fed UNCO had lower (*P* < 0.001) dCH_4_ concentrations than those fed control or UN. No significant interactions between treatment and sampling time were observed for any of the fermentation variables reported.

**Table 4:** Effect of urea plus nitrate pretreated rice straw (UN) and combination of UN and corn oil supplementation (UNCO) on dissolved gases and fermentation end products in the rumen of goats

Items	Treatment			Time after morning feeding			SEM	*P* - value		
	Control	UN	UNCO	0 h	2.5 h	6 h		Treatment	Time	Treatment × Time
pH	6.34^b^	6.47^a^	6.33^b^	6.51^a^	6.30^b^	6.34^b^	0.072	< 0.001	< 0.001	0.42
NH_4_^+^, mmol/L	8.26^b^	10.8^a^	11.2^a^	10.9	10.8	8.6	1.31	0.02	0.06	0.58
Dissolved gases										
Dissolved hydrogen, μmol/L	0.57^a^	0.44^b^	0.46^b^	0.30^c^	0.71^a^	0.46^b^	0.038	< 0.001	< 0.001	0.61
Dissolved methane, mmol/L	1.31^a^	1.23^a^	0.99^b^	1.55^a^	1.01^b^	0.96^b^	0.067	< 0.001	< 0.001	0.18
Total VFA, mmol/L	82.3	82.1	77.5	77.4^b^	85.6^a^	79.0^ab^	3.73	0.15	< 0.001	0.99
Molar proportion of individual VFA, mol/100 mol										
Acetate	70.1	70.5	69.2	69.7	70.4	69.7	0.89	0.19	0.57	0.97
Propionate	19.3	20.6	19.9	19.4	20.1	20.4	0.92	0.12	0.21	0.99
Butyrate	7.52^a^	6.15^b^	8.10^a^	7.50	6.91	7.36	0.472	< 0.001	0.28	0.72
Iso*-*butyrate	1.05^a^	0.90^b^	0.91^b^	1.16^a^	0.85^b^	0.84^b^	0.058	< 0.001	< 0.001	0.77
Valerate	0.65^a^	0.57^b^	0.65^a^	0.62^ab^	0.67^a^	0.59^b^	0.029	0.02	0.03	0.85
Iso-valerate	1.40	1.21	1.31	1.66^a^	1.13^b^	1.14^b^	0.102	0.09	< 0.001	0.96
Acetate/Propionate ratio	3.83	3.50	3.66	3.73	3.67	3.59	0.20	0.06	0.58	0.99
(Acetate + butyrate)/Propionate ratio	4.23^a^	3.82^b^	4.09^ab^	4.13	4.03	3.97	0.141	0.01	0.52	0.99

^a-c^Means with different superscripts within a row and heading are significantly different (*P* ≤ 0.05)

Goats fed UN had greater copies of protozoa (*P* < 0.001), methanogens (*P* < 0.001), *R. albus* (*P* < 0.05) and *S. ruminantium* (*P* < 0.001) in comparison with those fed control (Table 5). Corn oil supplementation to the UN diet decreased copies of protozoa (*P* < 0.001), methanogens (*P* < 0.001) and *S. ruminantium* (*P* < 0.001). Goats on all treatments had similar bacterial richness when expressed as Chao and Ace values and as Shannon-Weiner and Simpson indexes (Table 6). However, goats fed UN had greater abundance of Ruminococcaceae (*P* < 0.001) than those fed control, but supplementation of corn oil to the UN diet decreased the abundance of Ruminococcaceae (*P* < 0.001).

**Table 5:** Effect of urea plus nitrate pretreated rice straw (UN) and combination of UN and corn oil supplementation (UNCO) on selected groups of microorganisms (log_10_ copies/g DM rumen contents) in the rumen of goats

Items	Treatment			SEM	*P* - value
	Control	UN	UNCO		
Protozoa	10.6^b^	11.0^a^	10.4^b^	0.42	< 0.001
Fungi	10.4	10.5	10.7	0.14	0.13
Bacteria	13.7	13.8	13.6	0.10	0.36
Methanogens	12.0^b^	12.4^a^	12.1^b^	0.08	< 0.001
Selected groups of bacteria					
*Fibrobacter succinogenes*	10.9	11.1	10.9	0.13	0.13
*Ruminococcus albus*	9.98^b^	10.3^a^	10.1^ab^	0.13	0.047
*Ruminococcus flavefaciens*	10.2	10.1	10.1	0.10	0.34
*Selenomonas ruminantium*	12.1^b^	12.5^a^	12.1^b^	0.09	< 0.001

^a-b^Means with different superscripts within a row are significantly different (*P* ≤ 0.05)

**Table 6:** Effect of urea plus nitrate pretreated rice straw (UN) and combination of UN and corn oil supplementation (UNCO) on alpha diversity of bacterial community, and abundance of major phyla (relative abundance > 1%) and select families in the rumen of goats

Items	Treatment			SEM	*P* - value
	Control	UN	UNCO		
Alpha diversity index					
Number of OTU	36,693	33,587	35,882	4527	0.88
Shannon-Weiner index	4.02	4.04	3.59	0.339	0.38
Simpson index	0.07	0.07	0.12	0.018	0.08
Chao	786	716	734	79.2	0.74
Ace	777	703	729	77.4	0.71
Sobs	642	581	567	74.9	0.66
Relative abundances, %					
Bacteroidetes	64.8	54.2	61.2	3.52	0.12
Firmicutes	27.5	34.8	27.4	3.65	0.27
Ruminococcaceae	10.2^b^	14.7^a^	10.9^b^	1.28	< 0.001
Spirochaetae	1.25	2.47	1.40	0.357	0.06
Unclassified	2.14	1.72	1.24	0.711	0.69
Others	4.31	6.81	8.76	0.618	0.39

^a-b^Means with different superscripts within a row are significantly different (*P* ≤ 0.05)

## Discussion

### Feed digestibility

It has been reported that UN pretreatment improved ruminal degradation of rice straw measured in in vitro batch incubation [9]. The present in vivo study also indicates that goats fed UN had greater total-tract digestibility of NDF than those fed control. Urea in the UN pretreatment can be converted to ammonia during incubation with rice straw, which removes the polymerized silica-waxy compounds from the leaf fractions and destroys the covalent association between lignocellulose and exposes the inner tissues to bacterial colonization [28]. Increased in vivo fiber digestibility indicates that UN pretreatment allows greater access of ruminal fibrolytic bacteria to the fiber substrate. Griffith et al. [29] reported that ammoniation of fiber facilitates its degradation by rumen microbes. The rumen has a wide-range of fibrolytic microbial groups that utilize the fibrous substrates in feed. Protozoa and fungi are active fiber degraders, while other predominant fibrolytic bacteria include *R. albus*, *R. flavefaciens* and *F. succinogenes* [26]. In our study, UN pretreatment increased protozoa and *R. albus* populations, which may have contributed to increased total-tract digestibility of NDF in goats.

Although supplementation of corn oil to the UN diet did not further improve nutrient digestibility, fiber digestibility remained greater than that of the control treatment. It has been reported that high concentrations (> 40 g fat/kg DM) of fat supplementation exert negative effects on feed digestibility [30]. Thus, the relatively low dose of corn oil (30 g/kg DM) supplementation was not expected to decrease fiber digestibility. Similarly, Machmüller and Kreuzer [31] reported that 35 g oil /kg DM supplementation did not alter fiber digestibility in sheep. Although protozoa are major fiber degraders in the rumen [26], the decreased protozoa population observed for UNCO did not negatively affect fiber digestibility.

### Nitrogen balance

The greater N intake of goats fed UN did not alter total-tract digestibility of CP, but it increased rumen ammonia-N concentration and urinary N excretion, leading to a decrease in N retention. Djajanegara and Doyle [32] reported that urea pretreatment of rice straw increased fecal N excretion in sheep. Gunun et al. [5] reported that urea pretreated rice straw increased fecal and urinary N excretion in dairy cows. The urea and nitrate in the UN pretreatment provided additional non-protein N for rumen microbes. The lack of treatment effects on 16S rRNA gene copies of bacteria would suggest that the additional N supplied did not promote bacterial growth. It is likely that the soluble protein in the control diet was adequate for microbial growth, and additional N supplementation did not exert further benefits to the host animals. Furthermore, the greater observed protozoa population for the goats fed UN may have contributed to the decreased capture of ruminal N, resulting in increased ruminal ammonia concentration and urinary N excretion, because protozoa can prey on bacteria and reduce the amount of N used for bacterial protein synthesis [10].

Corn oil supplementation to the UN diet decreased N excretion and increased N retention. Benchaar et al. [33] reported that 40 g oil/kg DM reduced N excretion in dairy cows. Doranalli and Mutsvangwa [34] found that 60 g oil/kg DM decreased total N excretion and increased N retention in growing lambs. Improved N retention might be due to decreased intra-ruminal N recycling and increased microbial N flow to the duodenum [10]. In our study, corn oil supplementation to the UN diet decreased ruminal protozoa numbers, which has been reported to decrease predation of bacteria, and thus facilitate N capture by bacteria [10]. Therefore, supplementing corn oil to the UN diet offset the negative impact of UN pretreatment on N utilization, leading to an improvement of N retention in goats.

### Methane emission and rumen environment

Enteric CH_4_ emission was on average 9.9 ± 1.35 g/d, which is less than in other studies using goats or sheep. This discrepancy is likely due to the lower body weight (19.0 ± 1.22 kg) of goats used in the present study. Machmüller and Kreuzer [31] reported 29.4 g/d CH_4_ emissions in sheep with body weight of 78 ± 7.0 kg, while Abecia et al. [35] observed 15.9 g/d CH_4_ emissions in goats with average body weight of 43 ± 1.7 kg.

The UN treatment greatly decreased CH_4_ emissions in terms of g/kg DMI and g/kg OM digested, but did not alter daily CH_4_ emission in terms of g/d. This effect is due to the slight increase in DMI and OM digestibility in goats fed the UN diet. Nitrate acts as an alternative hydrogen sink and inhibits CH_4_ production in the rumen. When compared with control, the observed increase in protozoa and *S. ruminantium* populations for UN is consistent with the roles of these microorganisms in nitrate and nitrite reduction [27, 36]. Stoichiometrically, 4 mol of 2[H] can be redirected to ammonia away from methanogenesis during 1 mol of nitrate reduction to ammonia (NO_3_^−^ + 4 [2H] + 2H^+^ → NH_4_^+^ + 3H_2_O), equivalent to 258.1 g CH_4_/kg nitrate. If 2.33 g nitrate/kg dietary DM in UN was completely reduced to ammonia, CH_4_ reduction would have been 0.6 g/kg DMI. However, the observed CH_4_ reduction of 2.1 g/ kg DMI is far more than that theoretical reduction. Toxic effects of nitrate and nitrite have been reported for methanogens, but the dietary nitrate content (2.33 g/kg DM) in the present study is far less than that in published studies (6.7 and 5.3 g/kg DM, respectively) [7, 27], indicating that toxic effects may not have been the main cause of CH_4_ reduction for the UN treatment.

The shift of rumen fermentation may contribute to the CH_4_ reduction for the UN treatment. The decreased butyrate molar proportion and (acetate + butyrate) to propionate ratio of the goats fed UN is consistent with the decrease in CH_4_ emission of UN compared with control. Acetate and butyrate formation results in net [H] production, while propionate formation incorporates [H] [37]. A shortage of [H] would result in reduced methanogenesis. Our previous in vitro study found that urea plus nitrate pretreatment for rice straw decreased CH_4_ production with a reduction of acetate to propionate ratio [9]. van Zijderveld et al. [38] observed that nitrate supplementation caused CH_4_ inhibition, which was accompanied by increased propionate molar proportion at the expense of acetate and butyrate.

Oil supplementation inhibits CH_4_ production [39], due to a reduction in OM intake, rumen fermentation and protozoa associated methanogens. Guyader et al. [40] reported that a combination of 40 g linseed oil/kg dietary DM and 30 g calcium nitrate/kg dietary DM shows additive effects on CH_4_ mitigation. However, we did not find additive effects on CH_4_ mitigation when corn oil was supplemented to the UN diet. Therefore, the combination of UN and corn oil supplementation effectively decreases CH_4_ emissions, but not to a greater extent than UN alone.

As nitrate acts as an electron acceptor in anaerobic environments, reduction of nitrate to ammonia is associated with decreased H_2_ availability for methanogens [41]. However, nitrate or nitrite may exert toxic effect on methanogens, leading to an increase in ruminal H_2_ accumulation or dH_2_ concentration [39]. The balance between nitrate reduction and nitrite toxicity determines the available H_2_ in the rumen. In our study, UN treatment decreased ruminal dH_2_ concentration, indicating that nitrate in UN treatment incorporates [H] for ammonia synthesis during nitrate reduction. The low nitrate content (2.33 g/kg dietary DM) of the UN treatment was unlikely to have been toxic to the methanogens. Urea plus nitrate treatment did not alter dCH_4_ concentration, possibly because increased DMI and NDF digestibility can cause greater CH_4_ synthesis. When compared with control, goats fed UNCO had lower dCH_4_ concentration in rumen fluid, which is consistent with decreased CH_4_ emissions. However, in comparison with control, UNCO increased the fiber digestibility and ruminal ammonia concentration, but did not alter major individual VFA profile. These results are surprising and the underlying mechanism needs further investigation.

### Bacterial community

Urea plus nitrate treatment did not alter diversity of bacterial community, as shown by the similar Shannon-Weiner and Simpson indexes between UN and control groups. Bacteroidetes and Firmicutes are the two predominant phylums in the rumen bacterial community [42]. Although UN did not alter abundances of these phyla in comparison with control, the large increase in abundance of Ruminococcaceae family, which plays a very important role in fiber degradation [43], is consistent with the observed increase in fiber digestibility [44]. The greater 16S rRNA gene copies of *R. albus* of goats fed UN compared with control goats is consistent with the literature. Nguyen et al. [4] fed swamp buffaloes rice straw pretreated with urea plus limestone (20 + 20 g/kg DM of rice straw) and reported an increase in 16S rRNA gene copies of *R. albus*. It seems that the urea component in the UN treatment destroys the indigestible structural polysaccharide matrix, which facilitates colonization of fibrolytic bacteria by providing attachment sites for adherence of the bacteria. When corn oil was supplemented to the UN diet, abundance of Ruminococcaceae family was reduced, indicating that the unsaturated fatty acids in the oil had negative effects on microbiota of the rumen [45]. High-fat diets can decrease the abundance of Ruminococcaceae family [46]. When compared with control, UNCO did not alter bacterial diversity and abundance of major dominant bacteria and 16S rRNA gene copies of selected bacteria. Thus, it appears that corn oil supplementation can reverse the negative effects of UN on bacteria, resulting in a similar bacterial community as the control group.

## Conclusions

Urea plus nitrate pretreated rice straw increased total-tract fiber digestibility and decreased CH_4_ emissions but with decreased N retention. Greater fiber digestibility was associated with increased protozoa and *R. albus* populations and greater abundance of Ruminococcaceae. The urea in the UN pretreatment destroyed the covalent association between lignocellulose and exposed the inner tissues for attachment of fibrolytic microorganisms. Nitrate in the UN pretreatment appeared to incorporate [H] away from methanogenesis, leading to a reduction in ruminal dH_2_ concentration and methanogenesis. Supplementation of corn oil to the UN diet increased N retention, which may have been due to the decreased protozoa population. Compared with the control group, UNCO treatment increased fiber digestibility and decreased CH_4_ emissions, although most of the selected ruminal microorganisms were not changed. A combination of UN pretreatment and corn oil supplementation improved fiber digestibility and decreased CH_4_ emissions without affecting N balance, and thus may provide a potential strategy to increase nutritive value of rice straw for ruminant production.

## Abbreviations

[H]: Metabolic hydrogen
ADF: Acid detergent fiber expressed inclusive of residual ash
CH_4_: Methane
CP: Crude protein
dCH_4_: Dissolved methane
DE: Digestible energy
dH_2_: Dissolved hydrogen
DM: Dry matter
DMI: Dry matter intake
EE: Ether extract
*F. succinogenes*: *Fibrobacter succinogenes*
GE: Gross energy
N: Nitrogen
NDF: Neutral detergent fiber expressed inclusive of residual ash
OM: Organic matter
qPCR: Real-time quantitative polymerase chain reaction
*R. albus*: *Ruminococcus albus*
*R. flavefaciens*: *Ruminococcus flavefaciens*
*S. ruminantium*: *Selenomonas ruminantium*
UN: Urea plus nitrate treatment
UNCO: UN diet supplemented with corn oil
VFA: Volatile fatty acid
